# Diaqua­bis(pyrazine-2-carboxyl­ato-κ^2^ *N* ^1^,*O*)manganese(II) dihydrate

**DOI:** 10.1107/S1600536808006417

**Published:** 2008-03-14

**Authors:** Hui-Dong Xie, Cheng-Zhi Xie, Fang-Fang Dang

**Affiliations:** a School of Science, Xi’an University of Architecture and Technology, Xi’an 710055, People’s Republic of China; b Department of Chemistry, Luoyang Normal University, Luoyang 471022, People’s Republic of China

## Abstract

In the title compound, [Mn(C_5_H_3_N_2_O_2_)_2_(H_2_O)_2_]·2H_2_O, the Mn^II^ atom, lying on an inversion centre, has a distorted octa­hedral environment and the molecules are linked by O—H⋯O and N—H⋯O hydrogen bonds to form a three-dimensional supra­molecular structure.

## Related literature

For related literature, see: Ciurtin *et al.* (2002 ▶); Dong *et al.* (2000 ▶); Klein *et al.* (1982 ▶); O’Connor & Sinn (1981 ▶); Ptasiewicz-Bak *et al*. (1995 ▶).
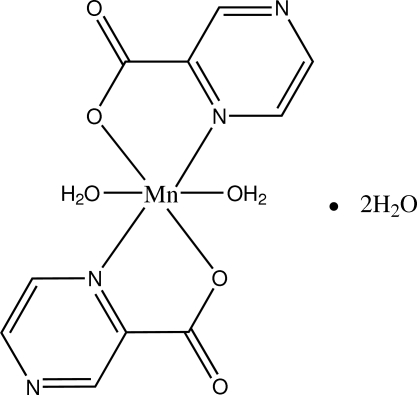


## Experimental

### 

#### Crystal data

[Mn(C_5_H_3_N_2_O_2_)_2_(H_2_O)_2_]·2H_2_O
*M* *_r_* = 373.19
Monoclinic, 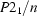

*a* = 7.233 (2) Å
*b* = 13.003 (4) Å
*c* = 8.257 (3) Å
β = 102.207 (5)°
*V* = 759.1 (4) Å^3^
*Z* = 2
Mo *K*α radiation
μ = 0.91 mm^−1^
*T* = 293 (2) K
0.20 × 0.10 × 0.10 mm

#### Data collection

Bruker SMART 1000 CCD area-detector diffractometer
Absorption correction: multi-scan (*SADABS*; Sheldrick, 2004 ▶) *T*_min_ = 0.838, *T*_max_ = 0.914
4297 measured reflections
1552 independent reflections
1252 reflections with *I* > 2σ(*I*)
*R*_int_ = 0.025

#### Refinement

*R*[*F* ^2^ > 2σ(*F* ^2^)] = 0.032
*wR*(*F* ^2^) = 0.081
*S* = 1.08
1552 reflections
106 parameters
H-atom parameters constrained
Δρ_max_ = 0.33 e Å^−3^
Δρ_min_ = −0.23 e Å^−3^

### 

Data collection: *SMART* (Bruker, 2001 ▶); cell refinement: *SMART*; data reduction: *SAINT* (Bruker, 2001 ▶); program(s) used to solve structure: *SHELXS97* (Sheldrick, 2008 ▶); program(s) used to refine structure: *SHELXL97* (Sheldrick, 2008 ▶); molecular graphics: *SHELXTL* (Sheldrick, 2008 ▶); software used to prepare material for publication: *SHELXTL*.

## Supplementary Material

Crystal structure: contains datablocks I, global. DOI: 10.1107/S1600536808006417/cs2069sup1.cif

Structure factors: contains datablocks I. DOI: 10.1107/S1600536808006417/cs2069Isup2.hkl

Additional supplementary materials:  crystallographic information; 3D view; checkCIF report

## Figures and Tables

**Table:** 

Mn1—O3	2.0670 (18)
Mn1—O1	2.0738 (16)
Mn1—N1	2.1246 (19)

**Table:** 

O3—Mn1—O1^i^	90.24 (7)
O3—Mn1—O1	89.76 (7)
O3—Mn1—N1^i^	91.51 (8)
O1—Mn1—N1^i^	101.65 (7)
O3—Mn1—N1	88.49 (8)
O1—Mn1—N1	78.35 (7)

Symmetry code: (i) 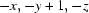.

**Table 2:** Hydrogen-bond geometry (Å, °)

*D*—H⋯*A*	*D*—H	H⋯*A*	*D*⋯*A*	*D*—H⋯*A*
O3—H3*B*⋯O4^ii^	0.85	1.79	2.639 (3)	176
O3—H3*A*⋯O2^iii^	0.85	1.87	2.715 (2)	171
O4—H4*A*⋯O2^iv^	0.85	1.98	2.806 (3)	164
O4—H4*B*⋯N2	0.85	2.03	2.865 (3)	170

Symmetry codes: (ii) 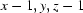; (iii) 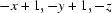; (iv) 
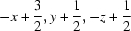
.

**Fig. 1.**
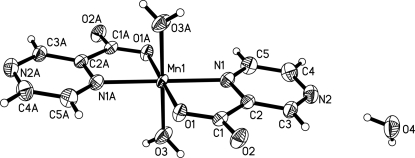
The structure of the title compound and the atomic numbering scheme, with atom labels and 35% probability displacement ellipsoids for non-H atoms (small spheres for the H atoms).

**Fig. 2.**
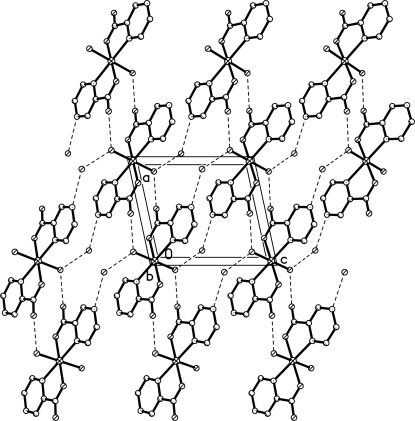
The packing of the title compound viewed down the b axis, showing the hydrogen bond donor-acceptor atoms. H atoms have been omitted for clarity.

## supplementary crystallographic information

### Comment

In the past decades, self-assembly processes involving metal ions and organic ligands directed by either metal coordination or hydrogen bonds have received a great deal of attention in the field of supramolecular chemistry and crystal engineering. Pyrazine carboxylic acids, containing O– or N– donors, are excellent bridging ligands when coordinated to transition metals and have been extensively studied as active ligands in the course of electron-transfer and magnetochemistry research (Klein *et al.*, 1982; O'Connor *et al.*, 1981). The cobalt(II), nickel(II), copper(II), zinc(II) and manganese(II) complexes of the 2-pyrazinecarboxylic acid ligand have been reported (Ciurtin *et al.*, 2002; Dong *et al.*, 2000; Ptasiewicz-Bak *et al.*, 1995). Ptasiewicz-Bak *et al.* reported an orthorhombic manganese(II) dipyrazinate dihydrate complex (space group *F*dd2), in which the coordination polyhedron around the Mn^II^ atom is a distorted octahedron with *cis* positioned water molecules. The title complex is another monomeric complex of Mn^II^ with the 2-pyrazinecarboxylic acid ligand, which is isostructrual to the cobalt(II) complex (Ptasiewicz-Bak *et al.*, 1995).

The Mn^II^ atom sits on an inversion center and the coordination geometry for the Mn^II^ atom (Fig. 1) is distorted octahedral (Table 1). Each Mn^II^ atom is axially coordinated by water molecules and consists of an equatorial plane of two oxygen donors and two nitrogen donors from two chelating 2-pyrazinecarboxylato group. As a consequence of the reaction the carboxylic groups of the starting diacid in position 3 are decarboxylated while the coordinated carboxylic groups in 2-position are kept and are deprotonated. The title molecules are connected by the O—H···N and O—H···O hydrogen-bonding interactions (Fig. 2); see Table 2 for the geometric parameters describing these interactions.

### Experimental

A mixture of manganese(II) chloride tetrahydrate, (0.4 mmol, 79.2 mg), pyrazine-2,3-dicarboxylic acid (0.8 mmol, 134.5 mg), and H_2_O (1.0 mol, 18.0 ml) in the molar ratio of 1: 2: 2500 was sealed in a 40 ml stainless steel reactor with Teflon liner and directly heated to 160 °C, kept at 160 °C for 72 h, and then directly cooled to the room temperature. Light-yellow block-shaped crystals of the title complex were collected by filtration and washed with ethanol (2×5 ml) for the structural analysis.

### Refinement

All H atoms were initially located in difference Fourier maps and were treated isotropically in the riding-model approximation with C—H = 0.93 Å, O—H = 0.85 Å, *U*_iso_(H) = 1.5*U*_eq_(O), and *U*_iso_(H) = 1.2*U*_eq_(C).

### Crystal data

**Table:**

[Mn(C_5_H_3_N_2_O_2_)_2_(H_2_O)_2_]·2H_2_O	*F*_000_ = 382
*M**_r_* = 373.19	*D*_x_ = 1.633 Mg m^−^^3^
Monoclinic, *P*2_1_/*n*	Mo *K*α radiation λ = 0.71073 Å
Hall symbol: -P 2yn	Cell parameters from 931 reflections
*a* = 7.233 (2) Å	θ = 3.0–26.4º
*b* = 13.003 (4) Å	µ = 0.91 mm^−^^1^
*c* = 8.257 (3) Å	*T* = 293 (2) K
β = 102.207 (5)º	Block, light-yellow
*V* = 759.1 (4) Å^3^	0.20 × 0.10 × 0.10 mm
*Z* = 2	

### Data collection

**Table:**

Bruker SMART 1000 CCD area-detector diffractometer	1552 independent reflections
Radiation source: fine-focus sealed tube	1252 reflections with *I* > 2σ(*I*)
Monochromator: graphite	*R*_int_ = 0.025
*T* = 293(2) K	θ_max_ = 26.4º
φ and ω scans	θ_min_ = 3.0º
Absorption correction: multi-scan(SADABS; Sheldrick, 2004)	*h* = −7→9
*T*_min_ = 0.838, *T*_max_ = 0.914	*k* = −16→15
4297 measured reflections	*l* = −10→9

### Refinement

**Table:**

Refinement on *F*^2^	Secondary atom site location: difference Fourier map
Least-squares matrix: full	Hydrogen site location: inferred from neighbouring sites
*R*[*F*^2^ > 2σ(*F*^2^)] = 0.032	H-atom parameters constrained
*wR*(*F*^2^) = 0.081	*w* = 1/[σ^2^(*F*_o_^2^) + (0.0336*P*)^2^ + 0.3749*P*] where *P* = (*F*_o_^2^ + 2*F*_c_^2^)/3
*S* = 1.08	(Δ/σ)_max_ < 0.001
1552 reflections	Δρ_max_ = 0.33 e Å^−^^3^
106 parameters	Δρ_min_ = −0.23 e Å^−^^3^
Primary atom site location: structure-invariant direct methods	Extinction correction: none

### Fractional atomic coordinates and isotropic or equivalent isotropic displacement parameters (Å^2^)

**Table:**

	*x*	*y*	*z*	*U*_iso_*/*U*_eq_	
Mn1	0.0000	0.5000	0.0000	0.02497 (15)	
C2	0.3799 (3)	0.50843 (18)	0.2061 (3)	0.0321 (5)	
O1	0.2234 (2)	0.40214 (13)	−0.0110 (2)	0.0374 (4)	
N1	0.2126 (3)	0.55522 (15)	0.1991 (2)	0.0344 (5)	
C1	0.3768 (3)	0.41836 (17)	0.0906 (3)	0.0316 (5)	
C5	0.2082 (4)	0.6331 (2)	0.3022 (3)	0.0449 (6)	
H5	0.0940	0.6661	0.3015	0.054*	
O2	0.5231 (2)	0.36634 (14)	0.1034 (2)	0.0461 (5)	
N2	0.5373 (3)	0.62300 (17)	0.4146 (3)	0.0457 (5)	
C3	0.5411 (3)	0.5443 (2)	0.3121 (3)	0.0388 (6)	
H3	0.6561	0.5123	0.3119	0.047*	
C4	0.3699 (4)	0.6661 (2)	0.4106 (3)	0.0495 (7)	
H4	0.3612	0.7200	0.4827	0.059*	
O3	0.0978 (2)	0.60221 (15)	−0.1543 (2)	0.0554 (5)	
H3A	0.2145	0.6136	−0.1497	0.083*	
H3B	0.0328	0.6326	−0.2386	0.083*	
O4	0.9068 (3)	0.69223 (17)	0.5751 (3)	0.0703 (7)	
H4B	0.7931	0.6798	0.5266	0.105*	
H4A	0.9341	0.7505	0.5398	0.105*	

### Atomic displacement parameters (Å^2^)

**Table:**

	*U*^11^	*U*^22^	*U*^33^	*U*^12^	*U*^13^	*U*^23^
Mn1	0.0141 (2)	0.0262 (2)	0.0330 (3)	0.00080 (19)	0.00120 (16)	0.0019 (2)
C2	0.0266 (10)	0.0339 (12)	0.0354 (12)	−0.0008 (10)	0.0058 (9)	0.0064 (10)
O1	0.0273 (8)	0.0382 (10)	0.0439 (10)	−0.0004 (7)	0.0009 (7)	−0.0047 (8)
N1	0.0285 (10)	0.0322 (11)	0.0419 (11)	0.0015 (8)	0.0061 (8)	0.0012 (9)
C1	0.0253 (11)	0.0315 (12)	0.0388 (13)	−0.0017 (9)	0.0081 (9)	0.0025 (10)
C5	0.0421 (15)	0.0396 (15)	0.0527 (16)	0.0052 (11)	0.0098 (12)	−0.0058 (12)
O2	0.0262 (9)	0.0475 (11)	0.0635 (12)	0.0075 (8)	0.0071 (8)	−0.0069 (9)
N2	0.0465 (13)	0.0424 (13)	0.0438 (13)	−0.0070 (10)	−0.0005 (10)	−0.0009 (10)
C3	0.0305 (12)	0.0406 (13)	0.0429 (14)	−0.0025 (10)	0.0022 (10)	0.0032 (12)
C4	0.0584 (17)	0.0384 (15)	0.0495 (16)	−0.0011 (13)	0.0066 (13)	−0.0080 (12)
O3	0.0276 (9)	0.0690 (14)	0.0664 (13)	−0.0023 (8)	0.0024 (9)	0.0293 (11)
O4	0.0569 (13)	0.0736 (16)	0.0679 (14)	−0.0166 (11)	−0.0148 (11)	0.0273 (12)

### Geometric parameters (Å, °)

**Table:**

Mn1—O3	2.0670 (18)	C1—O2	1.242 (3)
Mn1—O3^i^	2.0670 (18)	C5—C4	1.382 (4)
Mn1—O1^i^	2.0738 (16)	C5—H5	0.9300
Mn1—O1	2.0738 (16)	N2—C4	1.328 (4)
Mn1—N1^i^	2.1246 (19)	N2—C3	1.332 (3)
Mn1—N1	2.1246 (19)	C3—H3	0.9300
C2—N1	1.345 (3)	C4—H4	0.9300
C2—C3	1.383 (3)	O3—H3A	0.8500
C2—C1	1.507 (3)	O3—H3B	0.8500
O1—C1	1.259 (3)	O4—H4B	0.8500
N1—C5	1.328 (3)	O4—H4A	0.8501
			
O3—Mn1—O3^i^	180.0	C5—N1—Mn1	129.97 (17)
O3—Mn1—O1^i^	90.24 (7)	C2—N1—Mn1	112.20 (15)
O3^i^—Mn1—O1^i^	89.76 (7)	O2—C1—O1	125.5 (2)
O3—Mn1—O1	89.76 (7)	O2—C1—C2	118.1 (2)
O3^i^—Mn1—O1	90.24 (7)	O1—C1—C2	116.42 (19)
O1^i^—Mn1—O1	180.0	N1—C5—C4	121.3 (2)
O3—Mn1—N1^i^	91.51 (8)	N1—C5—H5	119.3
O3^i^—Mn1—N1^i^	88.49 (8)	C4—C5—H5	119.3
O1^i^—Mn1—N1^i^	78.35 (7)	C4—N2—C3	116.7 (2)
O1—Mn1—N1^i^	101.65 (7)	N2—C3—C2	122.2 (2)
O3—Mn1—N1	88.49 (8)	N2—C3—H3	118.9
O3^i^—Mn1—N1	91.51 (8)	C2—C3—H3	118.9
O1^i^—Mn1—N1	101.65 (7)	N2—C4—C5	121.9 (3)
O1—Mn1—N1	78.35 (7)	N2—C4—H4	119.1
N1^i^—Mn1—N1	180.00 (8)	C5—C4—H4	119.1
N1—C2—C3	120.4 (2)	Mn1—O3—H3A	123.4
N1—C2—C1	115.55 (19)	Mn1—O3—H3B	126.9
C3—C2—C1	124.1 (2)	H3A—O3—H3B	109.2
C1—O1—Mn1	116.93 (15)	H4B—O4—H4A	106.2
C5—N1—C2	117.5 (2)		

Symmetry codes: (i) −*x*, −*y*+1, −*z*.

### Hydrogen-bond geometry (Å, °)

**Table:**

*D*—H···*A*	*D*—H	H···*A*	*D*···*A*	*D*—H···*A*
O3—H3B···O4^ii^	0.85	1.79	2.639 (3)	176
O3—H3A···O2^iii^	0.85	1.87	2.715 (2)	171
O4—H4A···O2^iv^	0.85	1.98	2.806 (3)	164
O4—H4B···N2	0.85	2.03	2.865 (3)	170

Symmetry codes: (ii) *x*−1, *y*, *z*−1; (iii) −*x*+1, −*y*+1, −*z*; (iv) −*x*+3/2, *y*+1/2, −*z*+1/2.
